# Peripheral TDP-43 pathology in amyotrophic lateral sclerosis: toward a systemic proteinopathy

**DOI:** 10.1007/s00401-026-03086-3

**Published:** 2026-09-15

**Authors:** Philippe Codron, Marion Miranda, Maelle Garnier, Shiyang He, Julien Gouju, Julien Cassereau, Pascal Leblanc, Franck Letournel

**Affiliations:** 1 ALS Reference Center, Department of Neurology, University Hospital of Angers, Angers, France; 2 University of Angers, Inserm U1083-CNRS 6015, MITOVASC, SFR ICAT, Angers, France; 3 Neurobiology and Neuropathology, Department of Pathology, University Hospital of Angers, Angers, France; 4 Université Claude Bernard Lyon1, CNRS UMR 5261, INSERM U1315, Pathophysiology and Genetics of Neuron and Muscle (INMG-PGNM), Lyon, France; 5 University of Angers, MINT, UMR Inserm 1066, CNRS 6021, Angers, France

**Keywords:** Amyotrophic lateral sclerosis, TDP-43, Phosphorylated TDP-43, Peripheral pathology, Proteinopathy, Skeletal muscle, Biomarkers

## Abstract

Amyotrophic lateral sclerosis (ALS) is a fatal neurodegenerative disorder characterized by progressive degeneration of upper and lower motor neurons. Cytoplasmic accumulation of phosphorylated TAR DNA-binding protein 43 (pTDP-43) is the pathological hallmark of most ALS cases. While ALS has traditionally been viewed as a disease confined to the brain, spinal cord and motor nerves, recent studies have reported pTDP-43 pathology in multiple other tissues (skeletal and cardiac muscle, skin, minor salivary glands, gastrointestinal tract and lymph nodes), referred to as *peripheral pathology.* The detection of pTDP-43 beyond the nervous system suggests that ALS-associated TDP-43 proteinopathy may be more widespread than previously recognized and raises fundamental questions regarding the spatial and temporal landscape of ALS pathology. Peripheral pTDP-43 accumulation may reflect a systemic biological susceptibility affecting multiple tissues, propagation of pathological TDP-43 species between anatomical compartments, or a combination of both mechanisms. While its biological significance is yet to be determined, the presence of pTDP-43 in peripheral tissues broadens the current conceptual framework of ALS. It may also provide new opportunities for pathology-based biomarkers, therapeutic monitoring, and mechanistic studies aimed at understanding disease initiation and progression. However, current evidence is derived from small and methodologically heterogeneous cohorts, and peripheral pTDP-43 pathology is not restricted to ALS, emphasizing the need for larger standardized studies.

## Introduction

Amyotrophic lateral sclerosis (ALS) is a fatal neurodegenerative disorder affecting the motor neurons in the cerebral cortex, the brainstem and the spinal cord [18]. Patients develop progressive muscle weakness, dysarthria, dysphagia, and eventually restrictive respiratory failure, which remains the leading cause of death. The median survival time is 3–5 years from the onset of symptoms, and there is currently no curative treatment for the disease [11]. While 5–10% of cases are associated with a monogenic pathological mutation, the etiology of sporadic ALS cases remains unknown, likely involving complex interactions between genetic susceptibility, aging and environmental exposures [1, 53].

Understanding the pathophysiology of ALS is further complicated by the disease's marked clinical heterogeneity. Patients differ in terms of age at onset, pattern of motor involvement, rate of progression, and presence of non-motor signs, including cognitive, behavioral, metabolic, and autonomic manifestations [20, 28, 43, 61]. This variability complicates not only diagnosis, but also biomarker development, patient stratification, and therapeutic evaluation in research. More fundamentally, it raises the question of whether there are distinct pathophysiological pathways between patients and over time. However, most patients with ALS appear to share a common final neurodegenerative pathway: in sporadic cases and many genetic forms of the disease, cytosolic inclusions of TAR DNA-binding protein 43 (TDP-43) and its hyperphosphorylated form (pTDP-43) can be seen in neurons and glial cells, making it the defining pathological hallmark of ALS [3, 36, 37]. These inclusions have also been identified in patients with frontotemporal lobar degeneration (FTLD-TDP) and limbic-predominant age-related TDP-43 encephalopathy (LATE), broadening the spectrum of TDP-43-related proteinopathies [3, 35–37].

Since their identification as key players in disease, TDP-43 and pTDP-43 have greatly advanced our understanding of ALS biology and *post-mortem* disease diagnosis. Recent studies have expanded this framework further by demonstrating the presence of pTDP-43 outside the central nervous system (CNS) and the peripheral motor pathways conventionally involved in ALS, referred to here as *peripheral tissues*. These observations suggest that ALS-associated TDP-43 proteinopathy may have a broader systemic expression.

This narrative review summarizes the current evidence on peripheral pTDP-43 pathology in patients with ALS. It discusses how these findings may reshape the current concepts of ALS pathogenesis and explores their implications for disease mechanisms, biomarker development, and future therapeutic strategies.

## TDP-43 biology and pathological mechanisms in ALS

TDP-43, encoded by the *TARDBP* gene, is a ubiquitously expressed RNA- and DNA-binding protein belonging to the heterogeneous nuclear ribonucleoprotein family. Under physiological conditions, TDP-43 is primarily located in the nucleus but continuously shuttles between the nucleus and the cytoplasm. Through its interaction with thousands of RNA targets, TDP-43 plays a central role in RNA metabolism, including transcriptional regulation, alternative splicing (especially repression of cryptic exon inclusion), RNA transport, mRNA stability, microRNA biogenesis, and stress responses [48, 62].

In pathological conditions, TDP-43 undergoes a series of molecular alterations, including misfolding, nuclear depletion with cytoplasmic mislocalization, hyperphosphorylation, ubiquitination, cleavage into C-terminal fragments, and aggregation into insoluble inclusions [29, 40, 60]. These pathological aggregates are observed in the cytosol of neurons and glial cells in over 95% of sporadic ALS cases and many genetic forms of the disease, establishing TDP-43 and pTDP-43 inclusions as the pathological hallmark of ALS [3, 36, 37]. Notable exceptions include *SOD1*- and *FUS*-associated ALS cases, which typically lack TDP-43 pathology and instead exhibit aggregation of their respective disease proteins [31, 51, 63].

Current models suggest that TDP-43-mediated neurodegeneration results from a combination of loss-of-function and gain-of-toxic-function mechanisms [24]. Nuclear depletion impairs the physiological functions of TDP-43, especially RNA processing and cryptic exon repression, whereas cytoplasmic aggregation promotes cellular toxicity by disrupting RNA metabolism, proteostasis, nucleocytoplasmic transport, mitochondrial function, neuroinflammation and cell dynamics [29, 40, 60]. Another feature of misfolded TDP-43 is its ability to act as a template that induces conformational changes in nearby native TDP-43 molecules, a phenomenon referred to as prion-like seeding. Neuropathological studies have demonstrated that TDP-43 deposition follows a sequential pattern involving anatomically connected regions, which provides indirect support for prion-like propagation in the human nervous system [9]. This hypothesis is supported by animal studies demonstrating the propagation of TDP-43 pathology after intracerebral injection of TDP-43 strains in mouse models [45, 46]. In vitro studies have also shown that TDP-43 seeds can be intercellularly transmitted through the extracellular environment (direct release or within exosomes and microvesicles) or through cell-to-cell interactions via synaptic connections or nanotubes [17, 26, 27, 32, 39, 44, 52, 56]. There is increasing evidence that distinct TDP-43 conformational seeds may exist with variable degrees of toxicity and propagation properties, a concept also related to prion diseases and other proteinopathies [4, 32, 46].

## Evidence for peripheral pTDP-43 pathology in ALS

Over the past decade, studies have reported the presence of pathological protein inclusions in the peripheral tissues of patients with neurodegenerative diseases [67]. In particular, pTDP-43 pathology has recently been identified in several peripheral tissues of ALS patients using immunohistochemistry and immunofluorescence. These findings challenge the traditional neurocentric view of ALS, suggesting that TDP-43 proteinopathy is more widespread.

**Fig. 1:**
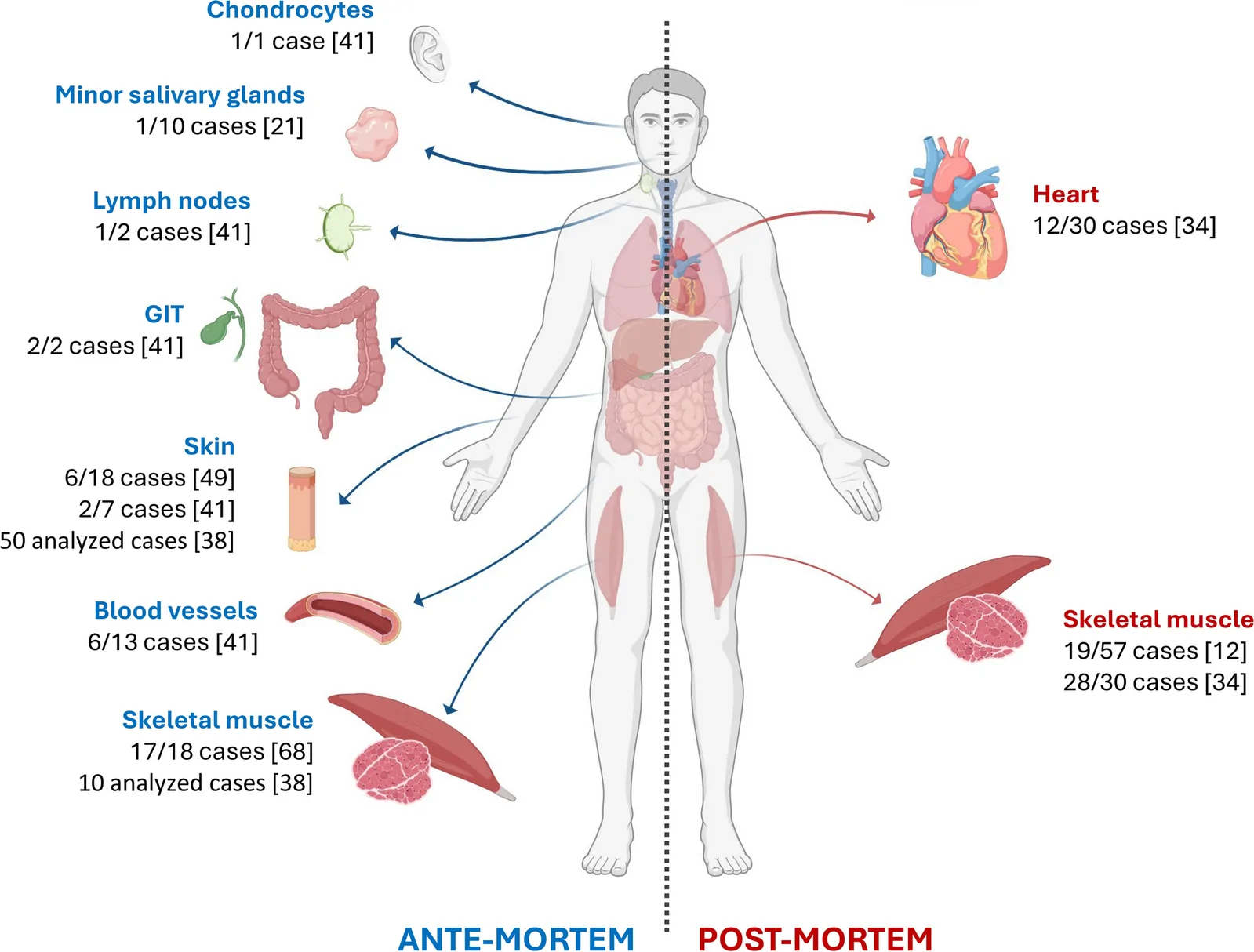
Distribution of reported peripheral pTDP-43 pathology in amyotrophic lateral sclerosis assessed by immunohistochemistry or immunofluorescence. Schematic representation of tissues in which phosphorylated TDP-43 (pTDP-43) pathology has been reported in patients with ALS. Blue labels indicate findings obtained from *ante-mortem* tissue samples, whereas red labels indicate findings from *post-mortem* analyses. Where available, ratios indicate the number of patients with pTDP-43 pathology relative to the total number of patients examined in each study. When a positivity rate was not reported, only the number of cases analyzed is indicated. *GIT* gastrointestinal tract

The results of studies on the peripheral detection of pTDP-43 are summarized below and in Table 1. Figure 1 summarizes the tissues in which the pathology has been identified, either *ante-mortem* or *post-mortem*.

**Table 1:** Histopathological studies investigating peripheral pTDP-43 pathology in ALS using immunohistochemistry or immunofluorescence

Tissue	Article	Country	Sampling	Studied tissue	ALS patients	Non-ALS control cases	Method	Anti-pTDP-43 antibody	Dilution	ALS positivity rate	Immunoreactivity in ALS	Control positivity rate	Immunoreactivity in Controls
Muscle	Cykowski et al., 2018 [12]	USA	*Post-mortem*	Axial (paraspinal and diaphragm) and appendicular (deltoid and quadriceps) muscle samples	57 ALS patients (diagnosis confirmed by autopsy) including 13 *C9*ALS patients	25 subjects: 8 paraspinal samples from laminectomy, and 17 muscle biopsies with neurogenic/myopathic changes, including 4 IBM	IHC	pSer409/410, rabbit polyclonal, Proteintech 22309-1-AP	1:500	19/57 (33.3%) ALS patients, including 4/13 C9ALS patients	Sarcoplasmic/subsarcolemmal aggregates (blocky, dot-, dash- and filament-like inclusions); no immunoreactivity in adjacent nerves	4/25 (16%) control subjects, all IBM (non-IBM 0/21)	Same inclusion morphologies, with lower burden compared to ALS patients
	Mori et al. 2019 [34]	Japan	*Post-mortem*	Tongue, cervical muscle, diaphragm, iliopsoas and myocardium samples	30 sporadic ALS patients (diagnosis confirmed by autopsy)	20 subjects: 13 subjects with NMDs (myositis, dystrophies, mitochondrial myopathies, congenital myopathies) and 7 without NMD	IHC	pSer409/410, rabbit polyclonal, Cosmo Bio	1:5000	30/30 (100%) ALS patients: 28/30 (93.3%) skeletal muscle and 12/30 (40%) myocardium samples	Sarcoplasmic aggregates: dense filamentous (round or stellate) and short-linear inclusions; dense forms associated with vacuolar degeneration; no immunoreactivity in adjacent nerves	12/20 (60%) control subjects (NMD: 9/13; non-NMD: 3/7)	Same inclusion morphologies, with lower burden compared to ALS patients
	Zhang et al. 2024 [68]	China	*Ante-mortem* (retrospective)	Archived biceps, deltoid, tibialis anterior and quadriceps biopsy samples	18 ALS patients (diagnosis based on the Airlie House criteria), including 3 *C9*ALS, 2 *SOD1*ALS and 1 *FUS*ALS patients	54 subjects with genetically or pathologically defined NMD	IHC, IF	pSer409/410, recombinant rabbit monoclonal, Proteintech 80007-1-RR	1:500	17/18 (94.4%) ALS patients including 3/3 *C9*ALS, 2/2 *SOD1*ALS, and 0/1 *FUS*ALS patients	Mainly subsarcolemmal, cytoplasmic and perinuclear aggregates	16/54 (29.6%) control subjects	Same inclusion morphologies, with lower burden compared to ALS patients
	Nolano et al. 2026 [38]	Italy	*Ante-mortem* (prospective)	Tongue biopsy samples	10 sporadic ALS patients (diagnosis based on the revised El Escorial criteria)	10 patients with idiopathic burning mouth syndrome	IF	pSer409/410, rabbit polyclonal, Proteintech 22309-1-AP	1:4000	NR as a case-level rate	pTDP-43 deposits in intramuscular nerve fascicles and neuromuscular junctions, including denervated endplates; granular, globular and dense linear aggregates in striated muscle fibers	NR as a case-level rate	Corresponding pathological changes absent in controls
Skin	Ren et al. 2018 [49]	China	*Ante-mortem* (prospective)	Distal-leg skin biopsy samples	18 sporadic ALS patients (diagnosis based on the revised El Escorial criteria)	18 age- and sex-matched subjects without neurological disease	IHC, IF	pSer409/410, mouse monoclonal (1A2C1), Proteintech 66318-1-Ig	1:50	6/18 (33.3%) ALS patients	pTDP-43 deposition in dermal autonomic nerve fibers; immunoreactivity in Meissner corpuscles, sweat glands and pilomotor innervation	1/18 (5.5%) control subjects	Immunoreactivity in Meissner corpuscles
	Pattle et al. 2023 [41]	United Kingdom	*Ante-mortem* (retrospective)	Archived skin surgical samples	7 ALS patients (diagnosis confirmed by autopsy)	No matched controls	IHC	pSer409/410, rabbit polyclonal, 2B Scientific CAC-TIP-PTD-M01	1:4000	2/7 (28.6%) ALS patients (2/8 skin biopsies)	pTDP-43 aggregates in superficial dermis, dendritic cells and peripheral nerve bundles/Schwann cells	NA	NA
	Nolano et al. 2026 [38]	Italy	*Ante-mortem* (prospective)	Hairy skin and glabrous fingertip skin biopsy samples	50 sporadic ALS patients (diagnosis based on the revised El Escorial criteria)	20 healthy controls and 20 patients with non-ALS neuropathy	IF	pSer409/410, rabbit polyclonal, Proteintech 22309-1-AP	1:4000	NR as a case-level rate	Increased pTDP-43 in keratinocytes, perivascular cells, arrector pili, smooth muscle cells, Schwann cells and Meissner corpuscles	NR as a case-level rate	Almost absent in healthy controls; low pTDP-43 levels in non-ALS neuropathy
MSG	Garnier et al. 2026 [21]	France	*Ante-mortem* (retrospective)	Archived minor salivary gland biopsy samples	10 ALS patients (diagnosis based on the Gold Coast criteria) including 1 *C9*ALS patient	20 patients without neurodegenerative disease	IHC	pSer409/410, rabbit polyclonal, Proteintech 22309-1-AP	1:6000	1/10 (10%) ALS patients	No aggregates but cytosolic pTDP-43 immunoreactivity in glandular cells and fibroblasts	0/20 control subjects	No pTDP-43 immunoreactivity detected
GIT	Pattle et al. 2023 [41]	United Kingdom	*Ante-mortem* (retrospective)	Archived colon and gallbladder surgical specimens	2 ALS patients (diagnosis confirmed by autopsy)	3 age- and sex-matched non-ALS colon biopsies	IHC	pSer409/410, rabbit polyclonal, 2B Scientific CAC-TIP-PTD-M01	1:4000	2/2 (100%) ALS patients	Cytoplasmic pTDP-43 dense perinuclear or dispersed dot-like aggregates in lamina propria	0/3 control subjects	No pTDP-43 immunoreactivity detected
Other tissues	Pattle et al. 2023 [41]	United Kingdom	*Ante-mortem* (retrospective)	Archived lymph nodes, soft tissue, auricular cartilage, gynaecological tissue, pleura, thyroid and urethra	13 ALS patients (diagnosis confirmed by autopsy)	No matched controls	IHC	pSer409/410, rabbit polyclonal, 2B Scientific CAC-TIP-PTD-M01	1:4000	6/13 (46.2%) ALS patients	pTDP-43 aggregates in peripheral vascular endothelial cells (6/13 patients), additional, non-exclusive involvement of lymph-node parenchyma (1/2 patients) and chondrocytes (1/1 patient)	NA	NA

*ALS* amyotrophic lateral sclerosis, *GIT* gastrointestinal tract, *IBM* inclusion body myositis, *IF* immunofluorescence, *IHC* immunohistochemistry, *MSG* minor salivary gland, *NMD* neuromuscular disease, *NA* Not applicable, *NR* not reported

### Skeletal and cardiac muscle

**Fig. 2:**
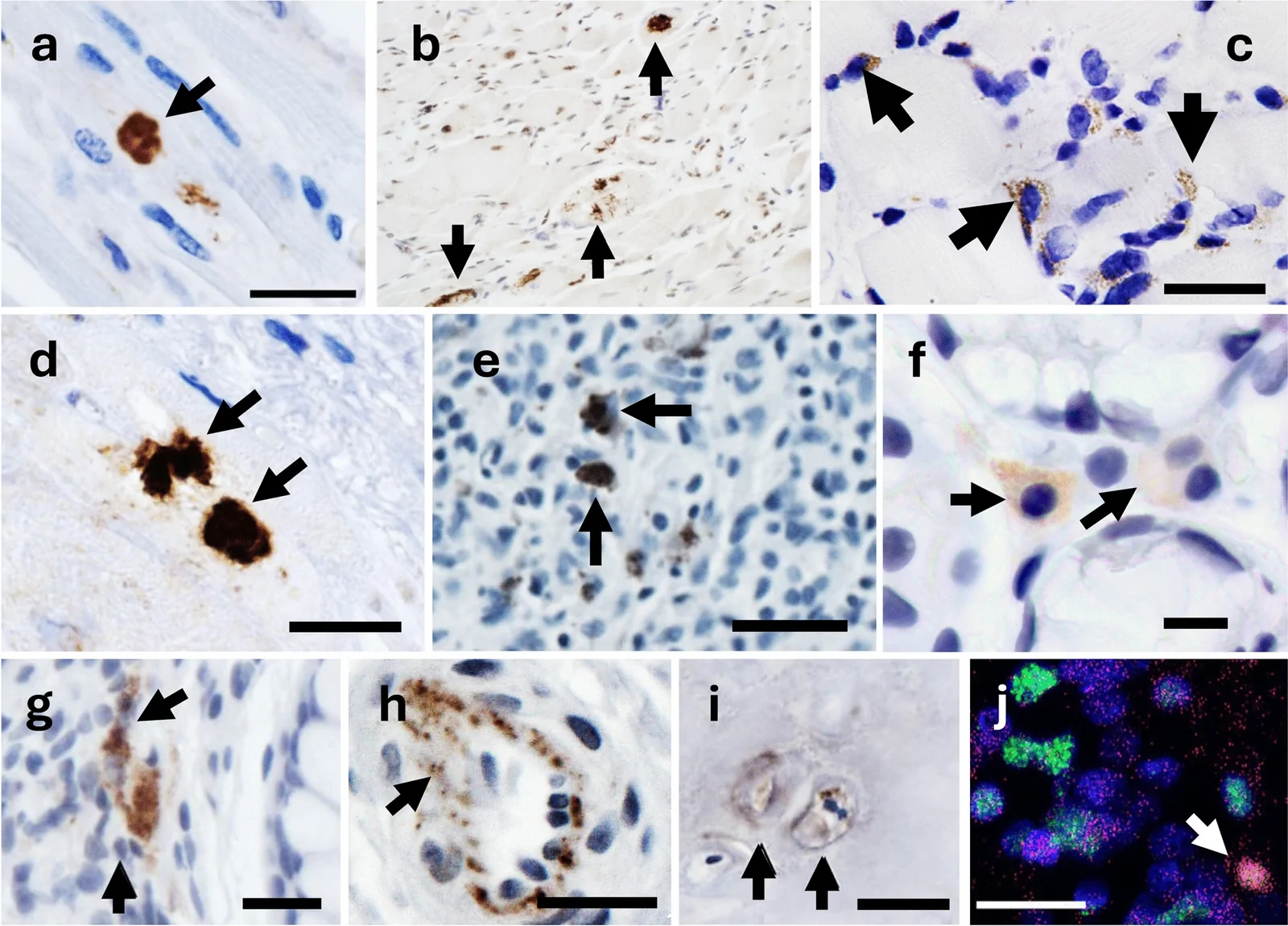
Illustrative pTDP-43 immunohistochemistry and immunofluorescence findings in peripheral tissues and retina of patients with ALS. **a** diaphragm, scale bar 20 μm [34]. **b** Paraspinous musculature, photographed at 400 × [12]. **c** Biceps, scale bar 20 μm [68]. **d** Myocardium, scale bar 20 μm [34]. **e** Superficial dermis, scale bar 40 μm [41]. **f** Minor salivary gland, scale bar 10 μm [21]. **g** Lamina propria of colonic tissue, scale bar 20 μm [41]. **h** Blood vessel, scale bar 10 μm [41]. **i** Chondrocytes of the ear, scale bar 10 μm [41]. **j** Retina (anatomically part of the central nervous system), scale bar 20 μm [42]. Representative micrographs reproduced or adapted from Cykowski et al. [12], Garnier et al. [21], Mori et al. [34], Pattle et al. [41], Pediconi et al. [42], and Zhang et al. [68], under the terms of their respective Creative Commons licenses

Skeletal muscle is the most extensively investigated peripheral tissue in ALS. Interest in muscle pathology stems from its close relationship with motor neurons and the neuromuscular junction. Cykowski et al. [12] identified pTDP-43 sarcoplasmic inclusions in approximately one-third of ALS autopsy cases, predominantly within axial muscles. Shortly thereafter, Mori et al. demonstrated a higher detection rate by identifying pathological inclusions in at least one muscle region in all ALS cases, including skeletal muscle in 28/30 (93.3%) cases and myocardium in 12/30 (40%) cases [34]. These aggregates displayed characteristic filamentous or linear morphologies (Fig. 2a–d). Zhang et al. [68] extended these observations to living patients by demonstrating pTDP-43 sarcoplasmic aggregates in 94.4% of routine muscle biopsies. Notably, pathological inclusions were identified in some individuals at very early stages of the disease, even in patients without clinical or electrophysiological abnormalities in the biopsied muscles. More recently, Nolano et al. extended these observations to *ante-mortem* tongue biopsies from 10 patients with ALS. pTDP-43 granular, globular, or dense linear aggregates were observed in striated muscle fibers, intramuscular nerve fascicles, and neuromuscular junctions [38].

### Skin

The skin has attracted considerable interest because it is easily accessible and can be repeatedly sampled during life. Ren et al. [49] identified pTDP-43 immunoreactivity in skin biopsies of approximately one-third of ALS patients, within autonomic nerve fibers and Meissner corpuscles. In addition, Pattle et al. identified pTDP-43 pathology in 2 of 8 archived *ante-mortem* skin specimens from seven patients who subsequently developed ALS. Inclusions were detected in the superficial dermis and peripheral nerve bundles up to several years before ALS diagnosis [41] (Fig. 2e). More recently, Nolano et al. provided direct evidence of pTDP-43 pathology in skin biopsies obtained *ante-mortem* from patients with ALS. pTDP-43 deposits involved multiple neural and non-neural cutaneous compartments, including keratinocytes, perivascular cells, smooth muscle structures, dermal nerve-associated cells, and Meissner corpuscles [38].

### Minor salivary glands

The minor salivary glands are another attractive biopsy site due to their accessibility and established use in clinical neurology. In the first study to examine this tissue in ALS patients in 2026, Garnier et al. detected pTDP-43 cytosolic immunoreactivity in the glandular cells and fibroblasts of a patient with advanced, rapidly progressive disease [21] (Fig. 2f). However, the detection rate was low (1/10 patients) and no aggregates were identified.

### Gastrointestinal tract

Pattle et al. reported pTDP-43 pathology in both available gastrointestinal specimens from patients who subsequently developed ALS. In colonic tissue, aggregates were identified in the lamina propria, including macrophages and dendritic cells, as well as neuronal cells within peripheral nerve bundles. In the gallbladder, pTDP-43 inclusions were found in neuronal and glial cells of the myenteric plexus, endothelial cells, macrophages, and dendritic cells. Both specimens had been obtained before ALS diagnosis, including one approximately one year before symptom onset [41] (Fig. 2g).

### Other peripheral tissues

In the same cohort, pTDP-43 inclusions were also identified in lymph-node parenchyma and vascular endothelial cells (Fig. 2h). In one patient, lymph-node pathology involved interfollicular/paracortical regions and was detected 14 years before ALS diagnosis. pTDP-43 aggregates were also observed in auricular chondrocytes in one patient [41] (Fig. 2i).

### The particular case of the retina

Although the retina is anatomically part of the central nervous system (CNS) and therefore cannot be considered a peripheral tissue, the presence of retinal pTDP-43 inclusions has been investigated as a marker of pathology extending beyond the brain. Pediconi et al. [42] demonstrated increased cytoplasmic immunoreactivity of pTDP-43 without aggregates in the retinal ganglion cells of ALS patients compared to control subjects, associated with neuronal degeneration and microglial activation (Fig. 2j). However, Dijkstra et al. failed to detect retinal pTDP-43 inclusions in two ALS donors despite confirming pathology in FTLD-TDP cases [13]. Likewise, Hart de Ruyter et al. found retinal pTDP-43 pathology in seven out of eight FTLD-TDP cases but not in the one ALS patient they analyzed [23].

## Critical appraisal of current evidence

### Temporal context of peripheral pTDP-43 pathology

Peripheral pTDP-43 pathology has been reported in tissue samples obtained before symptom onset or early in the clinical course of ALS. Importantly, however, matched brain and spinal cord tissue was not available at the time of peripheral sampling in these studies. These observations therefore cannot determine whether peripheral pathology precedes CNS involvement, but indicate that peripheral pTDP-43 may be detectable before overt neurological manifestations.

### Extent and detectability of peripheral pTDP-43 pathology

Current findings suggest that skeletal muscle is among the most consistently affected peripheral tissues in ALS, with protein aggregates similar to those observed in the nervous system. The spectrum of peripheral pTDP-43 pathology extends beyond skeletal muscle, but with a more heterogeneous prevalence. Furthermore, it may be confined to cytosolic pTDP-43 immunoreactivity without inclusions, as observed in the minor salivary glands or the retina, which does not carry the same biological significance. It is also important to note that peripheral pTDP-43 pathology has not been uniformly detected across all tissues examined: Pattle et al. reported no parenchymal pTDP-43 immunoreactivity in several *ante-mortem* samples, including thyroid, pleura, female reproductive tissues, urethra, and adipose tissue [41]. This result suggests that peripheral TDP-43 accumulation may affect specific cellular populations or tissues rather than being ubiquitous.

These apparent differences in detection should nevertheless be interpreted cautiously, given the limited sample sizes available for most tissues and the differences in populations and disease stages. There are also substantial differences in the antibody clones and dilutions used for pTDP-43 detection (Table 1), antigen retrieval procedures, tissue processing, and interpretation criteria. These methodological differences may considerably influence the detectability of pTDP-43 and should therefore be considered when comparing positivity rates across tissues or cohorts. Orthogonal validation of pTDP-43 immunoreactivity and blinded assessment are also rarely reported, which is particularly relevant for compartments prone to non-specific staining or autofluorescence. Finally, peripheral deposits have not yet been characterized biochemically or ultrastructurally, so that peripheral TDP-43 pathology currently remains an immunohistochemical observation rather than a fully characterized proteinopathy.

### Peripheral pTDP-43 pathology across ALS and other disorders

Peripheral pTDP-43 pathology is not restricted to ALS. Studies on muscle tissue have also demonstrated the presence of pTDP-43 inclusions in subjects with inclusion body myositis, polymyositis, myasthenia gravis, myotonic dystrophy, congenital myopathies, and mitochondrial diseases [12, 34, 68] (Table 1). Similar observations have been made in skin and retinal studies [13, 23, 49]. These findings suggest that peripheral pTDP-43 may be present in various neuromuscular and neurodegenerative conditions, though typically with a lower burden or different distribution than in ALS. An alternative and non-exclusive interpretation, at least in skeletal muscle, is that sarcoplasmic pTDP-43 partly reflects a stereotyped myofibre response to degeneration and regeneration, since TDP-43 physiologically assembles into amyloid-like, seed-competent myo-granules during normal muscle regeneration [65].

Further studies using harmonized tissue processing, standardized immunohistochemical protocols, and consensus criteria for pathological interpretation on large cohorts of ALS patients, control cases of ALS mimics, and healthy subjects are necessary to define the spatial and temporal characteristics of peripheral pTDP-43 pathology.

## Biological significance of peripheral pTDP-43 pathology

ALS has traditionally been viewed as a motor neurodegenerative disorder. However, patients may also experience non-motor manifestations such as cognitive and behavioral impairment, autonomic dysfunction, metabolic abnormalities, gastrointestinal disturbances and systemic inflammatory changes, suggesting that the disease extends beyond the motor system. For example, the identification of pTDP-43 pathology in skin nerve fibers and the myocardium can be considered in light of the growing recognition of sensory and autonomic dysfunction and cardiac involvement in ALS [6, 14, 33], although a causal relationship has yet to be established.

The detection of pTDP-43 in non-neuronal tissues raises important questions regarding the spatial and temporal extent of the disease. Whether peripheral pTDP-43 pathology reflects a widespread systemic proteinopathy arising across multiple tissues, or whether the disease emerges locally and subsequently propagates from or to the nervous system remains to be clarified. Furthermore, prion-like mechanisms and selective vulnerability of specific cell populations and tissues may both contribute to the propagation of the pathology.

### Peripheral pathology as a parallel manifestation of a systemic susceptibility

The presence of pTDP-43 deposits in tissue samples obtained months or years prior to the onset of neurological symptoms raises the possibility that peripheral pathology may be an early manifestation of a systemic process. Genetic and/or environmental factors that promote TDP-43 misfolding, hyperphosphorylation, aggregation, or impaired clearance could theoretically affect multiple tissues simultaneously, given that the protein is ubiquitously expressed. Activation of innate and adaptive immune pathways, together with TDP-43-related abnormalities reported in peripheral lymphocytes, could represent another manifestation of such systemic susceptibility [2, 47].

Differences in cellular resilience, regenerative capacity, and proteostatic mechanisms may subsequently determine which tissues ultimately develop overt pathology [16, 22, 57, 59]. In particular, post-mitotic cells, such as myofibers and neurons, may be more vulnerable to progressive proteotoxic stress and the accumulation of pathological TDP-43 compared with mitotically active cells in tissues such as skin or glands. This differential cellular vulnerability could hypothetically contribute to the heterogeneous distribution of peripheral pathology across tissues. ALS may therefore involve a broader biological process in which TDP-43 pathology emerges across multiple tissues but manifests most prominently within particularly susceptible tissues and neural networks.

Consistent with this possibility, Cykowski et al. observed pTDP-43-positive muscle fibers despite the absence of pTDP-43 inclusions in adjacent peripheral nerve elements, suggesting that muscle pathology may, at least in some cases, arise independently of local neuronal involvement [12]. Similarly, specific *TARDBP* mutations provide additional support for tissue-selective vulnerability. The p.W385IfsX10 and p.G376V variants, both located within the prion-like C-terminal domain of TDP-43, have been associated with primary myopathies characterized by prominent skeletal muscle TDP-43 aggregation without overt motor neuron degeneration [15, 69]. Although these observations are limited to a small number of variants and patients, they suggest that the phenotypic expression of TDP-43 proteinopathy may depend, at least in part, on tissue-specific effects of *TARDBP* mutations.

### Upstream or downstream propagation: nervous-system-first vs body-first models

An alternative explanation, though not mutually exclusive, is that pathological TDP-43 spreads progressively between anatomical compartments through interconnected networks. The detection of pTDP-43 in lower motor neuron fibers [30, 50], as well as in sensory and autonomic nerve fibers within peripheral tissues [38, 41, 49], provides indirect support for the hypothesis that TDP-43 pathology may propagate along axons, although the direction of this process cannot be determined. A conceptual framework inspired by propagation models proposed for other neurodegenerative diseases—particularly Parkinson's disease [7, 25]—could be proposed, although the evidence supporting an analogous model in ALS remains limited.

In a *nervous-system-first model*, TDP-43 pathology would originate in vulnerable neuronal populations of the central nervous system and subsequently propagate toward peripheral tissues through neuronal pathways. This hypothesis is compatible with the stereotypical progression of TDP-43 pathology described in ALS staging studies, and with experimental evidence supporting intercellular transmission of pathological TDP-43 species [8, 9, 45, 46, 54]***.*** In this framework, peripheral pathology would be a downstream consequence of the nervous system disease resulting from centrifugal propagation along nerves. The predominant peripheral localization of pTDP-43 aggregates within muscles would be related to the preferential motoneuronal involvement in ALS. In addition to direct axonal transport, extracellular vesicles have recently emerged as potential mediators of centrifugal pathological dissemination. Neuron-derived extracellular vesicles containing pathological TDP-43 species have been identified in biological fluids from patients with ALS [10, 26]. Although their contribution to peripheral TDP-43 deposition remains unknown, extracellular vesicles represent one potential mechanism for intercompartmental transfer of pathological protein species.

Conversely, a *body-first model* would propose that pathological TDP-43 initially arises in peripheral tissues before reaching the nervous system. These tissues are continuously exposed to environmental stressors, inflammatory signals, metabolic disturbances, and infectious agents, all of which can influence protein homeostasis. Furthermore, pTDP-43 has been identified in peripheral biopsies obtained at very early disease stages and, in some cases, before the appearance of overt neurological symptoms, although—as discussed above—these observations do not establish that peripheral pathology precedes CNS involvement. Muscle and the neuromuscular junction have therefore been proposed as candidate sites for early peripheral pathological changes [16, 19, 57, 59].

Importantly, both models may be influenced by selective cellular vulnerability. The appearance and progression of pathology may depend not only on the direction of propagation but also on the intrinsic susceptibility of specific cell populations to proteotoxic stress and to TDP-43 strain-specific effects [32].

### Toward an integrated model of pTDP-43 pathology in ALS

Current evidence supports that ALS-associated TDP-43 proteinopathy extends beyond the nervous system, with mechanisms that remain to be defined. Rather than representing competing explanations, the models may describe complementary aspects of a complex biological process. Systemic vulnerability may facilitate the emergence of pTDP-43 pathology in multiple tissues, while network-based propagation mechanisms could contribute to its amplification and dissemination over time. Future longitudinal studies integrating neuropathology, molecular biomarkers, seeding assays, extracellular vesicle analyses, and multimodal imaging are necessary to clarify the temporal sequence of these events and establish the contribution of peripheral pathology to ALS pathogenesis.

## Clinical implications of peripheral pTDP-43 pathology

The detection of pTDP-43 in peripheral tissues may have implications extending beyond neuropathological characterization. These findings provide new insights for biomarker development, disease monitoring, mechanistic investigations, and therapeutic intervention.

### Peripheral tissue biomarkers

One of the major challenges in ALS remains the absence of a robust biomarker directly reflecting the underlying molecular pathology. Current diagnostic approaches primarily rely on clinical evaluation, electrophysiological investigations, and the exclusion of alternative diagnoses [58]. The demonstration of pTDP-43 pathology in accessible peripheral tissues raises the possibility of developing tissue-based biomarkers that more directly reflect the underlying proteinopathy. Beyond diagnosis, such biomarkers could potentially contribute to molecular stratification in clinical trials and, if shown to change over time, to monitoring disease progression or treatment response.

Among investigated tissues, skin and minor salivary glands offer practical advantages because of their accessibility and suitability for repeated longitudinal sampling. Until recently, however, their biomarker potential was limited by low detection rates and substantial variability between studies. Nolano et al. [38] provided preliminary evidence suggesting that cutaneous pTDP-43 could be a potential biomarker. Nevertheless, independent replication in larger cohorts and longitudinal validation will be required before its clinical value can be established.

Skeletal muscle remains the most extensively investigated peripheral tissue and has yielded some of the highest reported detection rates [12, 34, 38, 68]. However, its greater invasiveness limits its suitability for repeated sampling. Moreover, pTDP-43 pathology in muscle is not restricted to ALS, having also been reported in several non-ALS neuromuscular conditions with positivity rates ranging from 16 to 60% across studies, although generally with a lower pathological burden. Sampling variability between muscle groups and the patchy distribution of inclusions may further influence detection rates and complicate its use as a routine biomarker.

Thus, despite increasingly promising results, particularly in skin and skeletal muscle, the mere presence of peripheral pTDP-43 is unlikely to constitute a stand-alone diagnostic biomarker. Future studies should therefore move beyond binary detection toward standardized pathological characterization and determine whether peripheral lesions exhibit reproducible morphological patterns, nuclear TDP-43 depletion, distinct biochemical or ultrastructural features, and tissue-specific phenotypes comparable to those characterized in CNS TDP-43 proteinopathies. Systematic *ante-* and *post-mortem* studies will also be required to better define the spatial and temporal distribution of peripheral pTDP-43 pathology, identify the most informative tissues and sampling sites, and optimize detection methods. Once these methodological foundations are established, multicenter prospective studies will be needed to assess reproducibility, diagnostic performance, prognostic value, and ultimately the clinical utility of peripheral pTDP-43 detection.

Beyond conventional pTDP-43 immunohistochemistry, emerging molecular approaches may substantially broaden the detection of peripheral pathology in ALS. Although phosphorylation-specific antibodies provide high pathological specificity, they may fail to detect earlier or alternative pathological TDP-43 species. Complementary approaches can instead interrogate additional aspects of TDP-43 proteinopathy, including seed-competent species, pathological TDP-43 detected using RNA aptamers, and loss of normal TDP-43 function through cryptic exon analysis. Seeding amplification assays, for example, exploit the prion-like templating properties of pathological TDP-43 to detect and amplify seed-competent species and have recently generated positive signals in the olfactory mucosa of 44% of patients with ALS [64]. Recent work by Waldron et al. further illustrates the potential of complementary molecular approaches: using a TDP-43 RNA aptamer together with STMN2 cryptic exon BaseScope™ analysis, the authors detected TDP-43 pathology in a greater proportion of previously examined skin and lymph-node samples than conventional pTDP-43 immunohistochemistry, and revealed pathology in skeletal muscle that had not been detected by antibody-based methods [66]. Together, these approaches indicate that peripheral TDP-43 pathology can be interrogated across multiple biological levels and may provide a complementary window into early disease biology and biomarker development. Nevertheless, their clinical value will need to be assessed alongside increasingly sensitive circulating molecular biomarkers, which offer the additional advantage of being less invasive [10, 55].

### Therapeutic perspectives

The identification of peripheral pTDP-43 pathology may also influence therapeutic development. Most current therapeutic strategies targeting TDP-43 have focused primarily on the nervous system. However, if ALS involves systemic TDP-43 proteinopathy, effective interventions may need to address pathological processes occurring beyond the nervous system. Several therapeutic approaches are currently under investigation, including antisense oligonucleotides, gene therapies, aggregation inhibitors, autophagy enhancers, immunotherapies, and strategies targeting nucleocytoplasmic transport. A better understanding of systemic TDP-43 seeding mechanisms may support the development of novel therapeutic strategies aimed at interrupting both the neuronal and the peripheral spread of pathological protein species.

## Outstanding questions

Despite the growing recognition of peripheral pTDP-43 pathology in ALS, several fundamental questions remain unresolved. First, the true prevalence and distribution of peripheral pathology across tissues, disease stages, and clinical phenotypes remain incompletely defined. Most available studies have involved relatively small cohorts and heterogeneous methodologies, making direct comparisons difficult. Second, the temporal relationship between central and peripheral pathology remains unknown. It is likely critical for understanding ALS pathogenesis to determine whether peripheral TDP-43 accumulation represents an early manifestation of a systemic process, a downstream consequence of nervous system degeneration, or a potential site of pathological initiation. Third, the biological significance of peripheral pTDP-43 pathology remains unclear. Whether pathological aggregates actively contribute to tissue dysfunction and clinical manifestations, or merely represent by-products of broader disease processes, has yet to be established. Large, multicenter, longitudinal studies using standardized pathological and molecular detection protocols in multiple sites, along with clinical, functional and imaging data will be required to establish the spatial and temporal landscape of peripheral pTDP-43 pathology and its pathophysiological consequences.

Another outstanding question is how genetic background influences peripheral TDP-43 proteinopathy. Whether the distribution, burden, and temporal dynamics of peripheral pathology differ between sporadic ALS and genetic forms associated with canonical TDP-43 pathology in the nervous system (e.g. *TARDBP*, *C9ORF72*, or *VCP*) remains largely unknown. The occurrence of primary muscle TDP-43 proteinopathy associated with specific *TARDBP* variants further highlights the potential influence of genotype on tissue-specific pathological expression [15, 16, 69]. Conversely, preliminary observations suggest that peripheral pTDP-43 aggregates may also occur in some genetic forms not typically associated with neuronal TDP-43 pathology, including *SOD1*-related ALS [68]. Finally, whether peripheral aggregation of mutant SOD1 or FUS proteins similarly occurs in their respective genetic forms remains unexplored. Comparative studies across ALS genotypes could therefore provide unique insights into the determinants of peripheral protein aggregation.

## Conclusion

The detection of pathological pTDP-43 in peripheral tissues has expanded the view of ALS as a disorder that extends beyond the nervous system. Nevertheless, important uncertainties remain regarding the true prevalence of peripheral pathology, its temporal relationship to nervous system involvement, and its contribution to disease pathogenesis. Defining the spatial and temporal landscape of peripheral pTDP-43 pathology is essential to better understanding disease initiation and progression, improving biomarker development, and identifying novel therapeutic opportunities that target fundamental ALS pathomechanisms.

## Data Availability

No datasets were generated or analysed during the current study.

## Abbreviations

ALS: Amyotrophic lateral sclerosis
CNS: Central nervous system
DNA: Deoxyribonucleic acid
FTLD-TDP: Frontotemporal lobar degeneration with TDP-43 pathology
FUS: Fused in sarcoma
mRNA: Messenger RNA
pTDP-43: Phosphorylated TAR DNA-binding protein 43
RNA: Ribonucleic acid
SOD1: Superoxide dismutase 1
TARDBP: TAR DNA-binding protein gene
TDP-43: TAR DNA-binding protein 43

## Declarations

### Methods

The objective of this review was to synthesize human studies evaluating TDP-43 pathology in peripheral tissues. The methodological framework was guided by the SANRA recommendations [5]. A structured search strategy using predefined keywords was applied to the MEDLINE/PubMed database until May 1, 2026. References from the studied articles and articles citing them were also considered for selection. Fourteen peer-reviewed original articles directly investigating peripheral or retinal TDP-43 pathology in ALS were identified [12, 13, 21, 23, 30, 34, 38, 41, 42, 47, 49, 50, 64, 68]. The recent preprint by Waldron et al. [66] was considered separately because of its direct relevance to emerging molecular detection approaches. Table 1 summarizes the seven core histopathological studies based on immunodetection of phosphorylated TDP-43. A total of 69 articles were included in the narrative synthesis. Illustration: Fig. 1 was created using BioRender.com.
